# Role of ligninolytic enzymes of white rot fungi (*Pleurotus* spp.) grown with azo dyes

**DOI:** 10.1186/s40064-016-3156-7

**Published:** 2016-09-05

**Authors:** Prashant D. Kunjadia, Gaurav V. Sanghvi, Anju P. Kunjadia, Pratap N. Mukhopadhyay, Gaurav S. Dave

**Affiliations:** 1 Department of Biochemistry, Faculty of Science, The. M. S. University of Baroda, Vadodara, 390001 Gujarat India; 2 Max Planck Institute of Developmental Biology, Tubingen, Germany; 3 Ashok & Rita Patel Institute of Integrated Study and Research in Biotechnology and Allied Sciences, New Vallabh Vidyangar, 388021 India; 4 Department of Biotechnology, GeneOmbio Technologies, Krishna Chambers, Pune, India; 5 Department of Biochemistry, Saurashtra University, Rajkot, 360005 India

**Keywords:** Ligninolytic enzymes, Submerged fermentation, Thin layer chromatography, *Pleurotus* species

## Abstract

**Background:**

Total three *Pleurotus* species (*P. ostreatus*, *P. sapidus*, *P. florida*) was compared for ligninolytic enzyme production grown with Coralene Golden Yellow, Coralene Navy Blue and Coralene Dark Red azo dyes in liquid medium under shaking condition.

**Results:**

The biodegradation competency varied from species to species and it was found that *P. ostreatus*, *P. sapidus* and *P. florida* to 20 ppm dye concentration shows 88, 92 and 98 % decolorization, respectively for all three dyes. Production pattern of laccase, manganese dependent peroxidase and lignin peroxidase were studied during the growth of the organisms for 10 days. Laccase was found to be the major extracellular ligninolytic enzyme produced by fungus with negligible detection of lignin peroxidases. In all concentration of three dye studied, maximum laccase activity was observed on day 8, for 20 mg/l of dye laccase specific activity was 1–1.58 U/mg in *P. ostreatus,* 0.5–0.78 U/mg in *P. sapidus* and 1–1.92 U/mg in *P. florida*. Different factors (dye concentration, pH, protein and sugar estimation) influencing the ability of *Pleurotus* species to degrade dyes is documented and degradation was attributed to microbial action irrespective of pH change. HPTLC analysis of samples indicated degradation of dyes into intermediate products.

**Conclusion:**

Level of ligninolytic enzymes is playing a major role in degradation of dye, which is dependent on time of incubation and species of fungi.

## Background

The technical textiles market, in terms of volume, is projected to reach 42.20 Million Metric Tons by 2020, at a CAGR of around 4.68 % from 2015 to 2020 (Www.Marketsandmarkets.Com 2015). Unfortunately, around 30 % of the applied reactive dyes are wasted because of the dye hydrolysis in the alkaline dye bath, as a result dye effluents contain 0.6–0.8 g/l of dye (Beydilli et al. 1998; Vandevivere et al. 1998). It is estimated, around 15 % of the dyestuff is released into wastewater effluent by textile industries (Pearce et al. 2003). Some azo dyes released in wastewater are either toxic or can be modified biologically to toxic or carcinogenic compounds (Ventura-Camargo and Marin-Morales 2013). Advancement in scientific development in dye technology, synthesized dye are chemically and photolytically more stable and resistant to degradation in nature (Muhd Julkapli et al. 2014). The implementation of environment protection legislation, which controls the discharge of colored water and increase awareness of negative environmental impact of dyestuffs, resulted in an increasing number of studies on the biodegradation of dyes in recent years (Dos Santos et al. 2004; Kunjadia et al. 2012).

As synthesized dyes are relatively recalcitrant in nature, dye wastewater is usually treated by physicochemical processes like adsorption, membrane-filtration, physical–chemical flocculation combined with flotation, ion exchange, precipitation and ozonation (Muhd Julkapli et al. 2014). However, these technologies are costly, little adaptable to a wide range of dye wastewaters and usually inefficient for complete mineralisation of dyes (Fu and Viraraghavan 2002; Vandevivere et al. 1998; Zilly et al. 2002). To mitigate these recalcitrant pollutants, biological treatment or biodegradation is an environment friendly and cost-effective alternative to these technologies (Gueu et al. 2007). The use of ligninolytic fungi are one of the possible alternative, studied for the biodegradation of dyes. Perusal of literature demonstrates the potential of white rot fungi to degrade pollutants by producing extracellular ligninolytic enzymes (Kunjadia et al. 2012; Wen et al. 2010) and most of them have been focused on dye degradation and decolorization (Champagne et al. 2010). It is turning into a promising alternative to replace or supplement present treatment processes (Coulibaly et al. 2003; Fu and Viraraghavan 2002). Previously, Fu and Viraraghavan (2002) summarized fungal decolorization of dyes used in textile industries, they have reported on progress, mechanisms and factors affecting the process of dye degradation (Fu and Viraraghavan 2002).

The initial recognition of white rot fungi competency in decolorization lays the foundation for its application in dye degradation. With the same interest, present study focuses on exploration of dye degradation efficiency of three different *Pleurotus* sp.

From extremely diverse range of the textile dyes, most unanimously used three azo disperse dyes (CGY, CNB, CDR) have been opted in the present study. Main objectives of the experiment were: (i) To evaluate the potential of all three *Pleurotus* sp. in degradation of textile dyes (ii) Evaluation of different parameters i.e. pH, activity of ligninolytic enzymes, protein and sugar content during degradation of dyes (iii) Analysis of resultant products by HPTLC.

## Methods

### Fungal cultures

*Pleurotus ostreatus* (MTCC142) was procured from The Microbial Type Culture Collection, Institute of Microbial Technology (IMTECH), Chandigarh, India. *Pleurotus sapidus* and *Pleurotus florida* was procured from Directorate of Mushroom Research, ICAR, Solan, Himachal Pradesh, India. Cultures were maintained on YDA (Yeast Dextrose Agar) slants and plates at 25 °C by sub culturing every 30 day interval.

### Dye samples

Three dye samples Coralene Golden Yellow (2GN) (λ_max_ = 440 nm), Coralene Dark Red (λ_max_ = 475 nm) & Coralene Navy Blue (3G) (λ_max_ = 540 nm) were collected in form of powder from Ganesh Laxmi Textile mill, Surat, Gujarat, India.

### Inoculum preparation and degradation of dye in liquid medium

Mycelial culture of *P. ostreatus*, *P. florida & P. sapidus* were grown on Yeast Dextrose Agar (YDA) medium. Cultures were grown at 25 °C in submerged liquid cultures in Yeast Dextrose Broth Medium. 50 ml of the medium in 250 ml flasks was inoculated by 5 mm diameter mycelial agar plugs taken from the YDA plates. The inoculated flasks were kept on rotary shaker at 150 rpm at room temperature (RT) for 6 days. On 6th day, dye was added to final concentrations of 20 ppm (20 mg/l), 50 ppm (50 mg/l), 100 ppm (100 mg/l) and 200 ppm (200 mg/l) in each different flasks from the stock solution of 40 gm/l. Negative control was kept by taking 20 ppm (20 mg/l) concentration of each dye in 50 ml of YDB and positive control was kept as fungal culture without any dye in 50 ml of YDB. Time of dye addition was considered as 0 day for experimental conditions.

### Degradation of dye

The final concentration of each dye CGY, CNB and CDR in the medium on day 0 was considered to be 100 %. Changes in CGY, CDR & CNB each dye concentration was monitored by measuring O.D. at λ_max_ 440, 475 and 540 nm, respectively and pH was monitored at every 2 days interval up to 12 days (Yatome et al. 1993).

### Estimation of reducing sugar and total protein content

Reducing sugar was estimated by Dinitrosalicylic acid method (Miller 1959). Protein concentration was estimated by the method of Lowry et al. (1951).

### Ligninolytic enzyme assays

#### Laccase assay

Laccase enzyme was estimated using 0.1 ml 10 mM Guaiacol prepared in ethanol, 0.4 ml 50 mM Phosphate buffer (pH–5.0) and 0.5 ml enzyme solution, which was collected freshly from all the flasks and centrifuged at 10,000 rpm for 20 min. O.D. was recorded at 460 nm immediately after addition of enzyme solution and after 10 min of incubation at 30 °C (Arora and Sandhu 1985).

#### Lignin Peroxidase assay

LiP assay mixer contained 50 mM sodium tartarate buffer pH 3.0, Azure B dye 32, 100 µM hydrogen peroxide and 0.1 ml enzyme solution, which was collected freshly from all the flasks and centrifuged at 10,000 rpm for 20 min (Arora and Gill 2001).

#### Manganese dependent assay

Reaction mixture for MnP assay contains 0.9 ml 0.3 mM MnCl_2_ in 50 mM Sodium lactate Buffer (pH–5.0), 0.5 ml 40 μM H_2_O_2_ and 0.1 ml enzyme solution, centrifuged at 10,000 rpm for 20 min. O.D. was recorded at 610 nm immediately after addition of enzyme solution and after 10 min of incubation at 30 °C (Orth et al. 1993).

### HPTLC (high performance thin layer chromatography) of decolorized product

Alumina TLC plate pre-coated with silica gel 60 F254 (Merck KGaA, Germany) was used throughout the experiments for separation of dye constituents. 10 cm × 10 cm plate was used and 10 samples were loaded carefully. Samples were separated using solvent system of n-Butanol:distilled water:glacial acetic acid (60:30:10). Plates were dried and spectra were recorded at different wavelengths.

### Statistical analysis

Statistical analysis was performed using Microsoft Excel 2010 software and data were expressed as a mean ± SE. Results were analyzed by one way ANOVA and Student’s *t* test (significance at *P* < 0.05).

## Results and discussion

Fungi offers cheaper and efficient alternative for decolorization or degradation of recalcitrant textile dyes. In the present study, white rot fungi *P. ostreatus*, *P. sapidus* and *P. florida* were tested for ligninolytic enzyme activity and its role in dye degradation. Ligninolytic enzyme and dye degradation activity was tested against azo textile disperse dyes CGY, CNB and CDR in concentrations of 50, 100 and 200 (mg/ml). Extracellular ligninolytic enzyme assays, protein concentration, sugar estimation and pH measurement were analyzed. Further, HPTLC analysis was carried out for azo dyes and generated products. All the three *Pleurotus* species efficiently decolorized all three dyes viz. CGY, CNB, CDR. Decolorization percentage clearly showed extensive removal of CGY by *P. ostreatus*. However, *P. florida* showed more than 95 % of decolorization efficiency of all the three dyes. Degradation of dye in the present study can be attributed by biosorption or bioadsorotion process, biosoprtion is reported to be primarily process in wood rot fungi (Balan and Monteiro 2001; Fu and Viraraghavan 2002). Our results are in support of Balan and Monterio (2001) findings, indicating indigo dye decolorization by fungal adsorption and extracelluar degradation. Bioadsorption in present study has been linked to electrostatic pull between negative charged dyes and positively charged cell wall components of fungi (Aksu and Tezer 2000). Earlier, published reports on azo dye degradation by *P. ostreatus* are in accordance to our results of biodegradation of textile azo dyes (Andrade et al. 2013; Kalmış et al. 2007; Yesilada et al. 2003).

### Factors influencing dye degradation

#### Concentration of dye

**Fig. 1:**
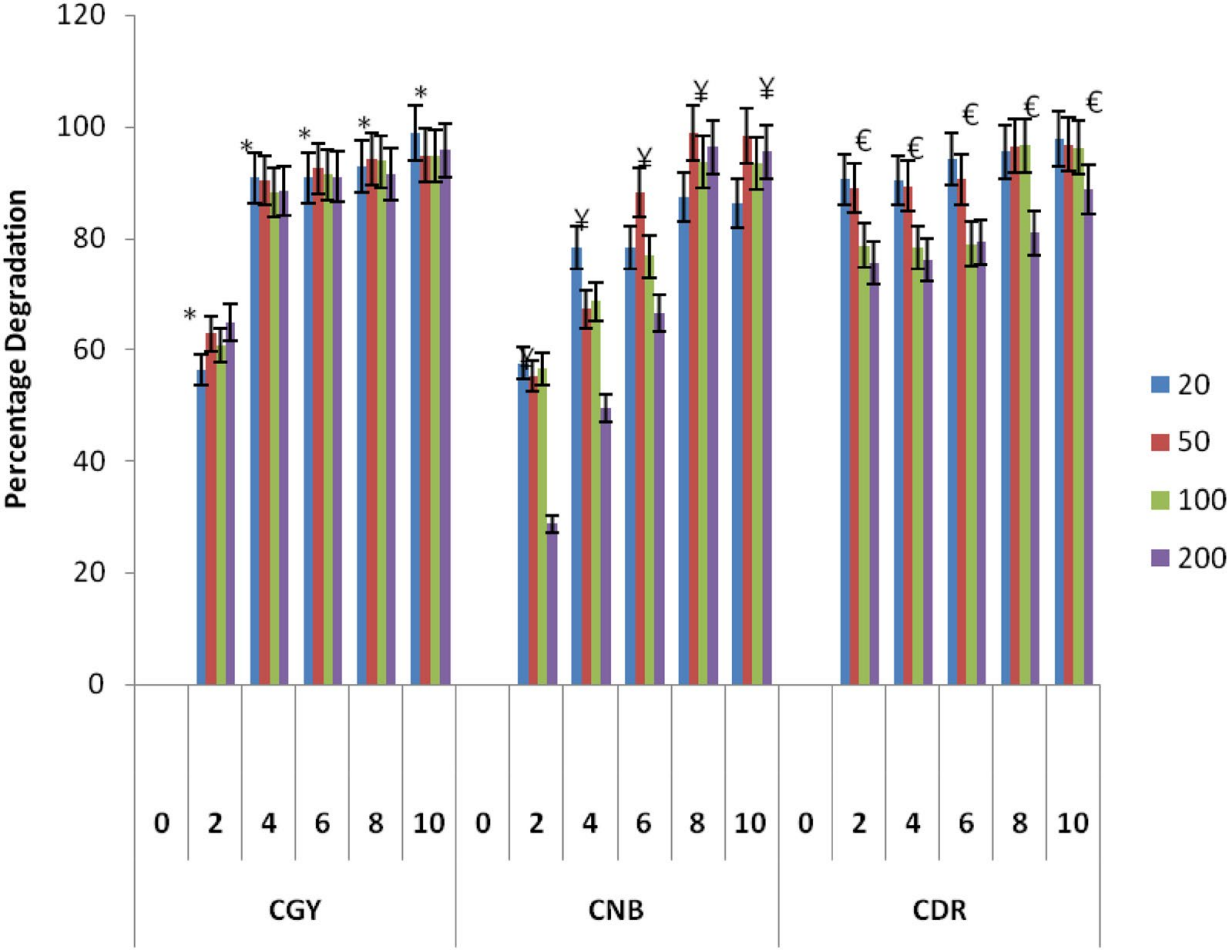
Dye decolorization by *P. florida*: CGY, CNB and CDR. *P < 0.05 compared to control group of CGY, ^¥^ *P* < 0.05 compared to control group of CNB, ^€^ *P* < 0.05 compared to control group of CDR

Among all the three species of *Pleurotus* taken for the study, *P. florida* showed maximum dye decolorization of all three textile azo dyes tested. *P. florida* showed maximum decolorization of CGY 98.9 %, whereas, *P. ostreatus* and *P. sapidus* showed 78.4 and 92 % decolorization in 20 ppm dye containing flask respectively. *P. florida* in general for all dyes with 20 ppm concentration showed more than 95 % of decolorization with CDR 97.9 %, CNB dye 98.3 % in 20 ppm dye containing flask (Fig. 1). In comparison between *P. ostreatus and P. sapidus* showed decolorization of CNB 89 and 90.7 %, CDR 88.1 and 91.4 % in the 20 ppm dye containing flasks respectively. It was worth to mention that *P. florida* even exhibited more than 90 % of decolorization efficiency in 50 ppm dye concentration whereas the other two *Pleurotus* species demonstrated decolorization 60–75 % of all the dyes in 50 ppm. Remarkably, total decolorization efficiency was found to be decreased above 100 ppm concentration. Decrement in decolorization efficiency at higher concentration due to factors like toxicity of dyes and inhibition of nucleic acid biosynthesis which ultimately inhibit cell growth (Chen et al. 2008; Radha et al. 2005).

Previous report showed decolorization potential of *Pleurotus* sp. using reference azo dye DB14 up to 400 mg/l (Singh et al. 2013). Above fact represents nature of dyes used and the possible binding sites available for the uptake of dyes. It is well documented fact that biodegradation of azo dyes takes place only upon reduction of azo linkage with electrons from co-substrate (Sponza and Işık 2004). In this context, the dye decolorization of CGY, CNB and CDR at 50 mg/l concentration is significant.

#### Influence of pH

The effect of initial pH on dye decolorization by fungi varied depending on the type of the dye. In the present study, initial pH of the aqueous solution of the dyes was kept in range of 5–7. Percentage removal of dye increased with increase in time irrespective of pH. Maximum removal of dye was observed at pH range of 6–6.5. Further, increase or decrease in pH decreased the decolorization of dye. Optimum pH for the color removal by white rot fungi was often at a neutral or slightly alkaline pH and the rate of color removal tended to decrease rapidly under strongly acid or strongly alkaline conditions, without any relationship to dye structure (Pearce et al. 2003). Previous reports suggest that interaction between sorbent and dye molecules is affected by the pH of the dye solution in different ways. Firstly, as dyes are complex aromatic organic compounds with different functional groups and unsaturated bonds, they have different ionization potentials at different pH, resulting in the pH dependent net charge on dye molecules. Secondly, surface of the biosorbent consists of biopolymers with many functional groups, so net charge on biosorbent measured in the form of zeta potential, is also pH dependent (Maurya et al. 2006). The effect of pH on the sorption of metals has been reported in detail elsewhere (Greene et al. 1987; Schiewer and Volesky 1995; Schiewer and Wong 2000; Veglio and Beolchini 1997). Low pH favor adsorption of dyes (Aksu and Dönmez 2003) and heavy metals (Sb and Abraham 2001) by the biomass of fungi and also by other adsorbents such as eucalyptus bark (Morais et al. 1999).

### Ligninolytic enzyme profiles

**Fig. 2:**
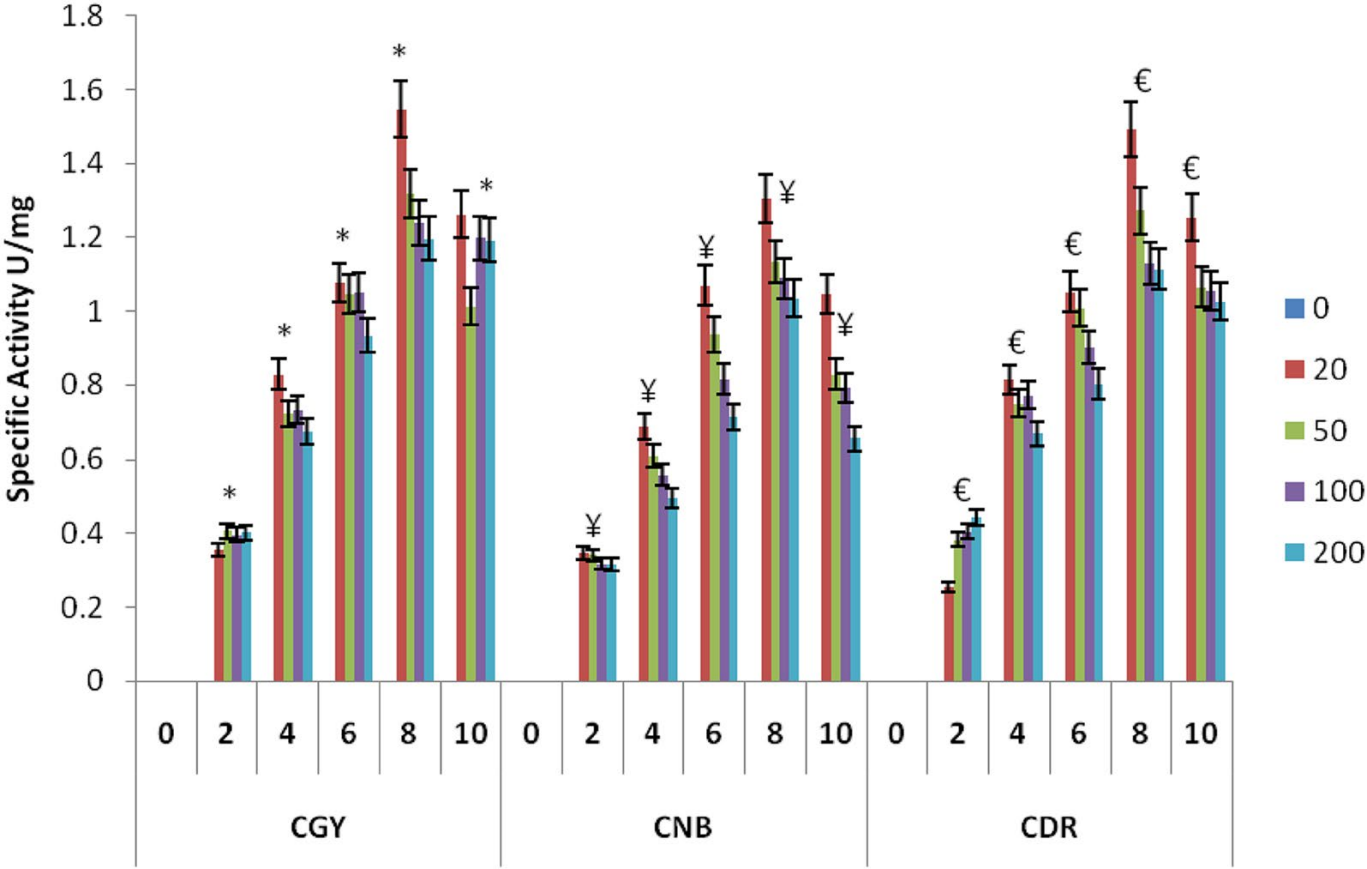
Laccase specific activity by *P. ostreatus*: CGY, CNB and CDR. *P < 0.05 compared to control group of CGY, ^¥^ *P* < 0.05 compared to control group of CNB, ^€^ *P* < 0.05 compared to control group of CDR

**Fig. 3:**
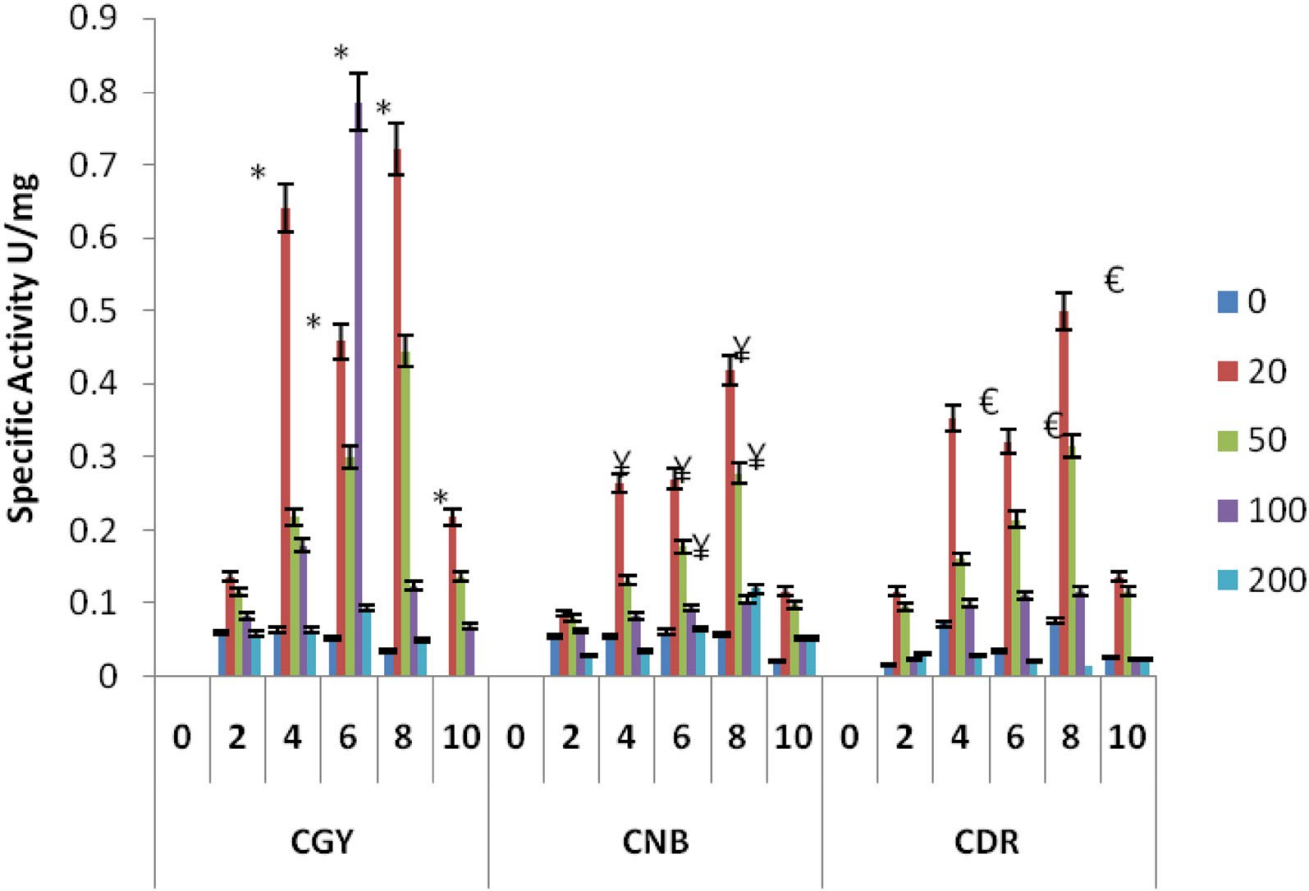
Laccase specific activity by *P. sapidus*: CGY, CNB and CDR. *P < 0.05 compared to control group of CGY, ^¥^ *P* < 0.05 compared to control group of CNB, ^€^ *P* < 0.05 compared to control group of CDR

Dye degradation by fungal cultures is often correlated to ligninolytic enzyme activities (Pointing 2001; Selvam et al. 2003). Several studies have been demonstrated the ability of fungal biomass and purified enzymes to decolorize dye (Wesenberg et al. 2003). In the present study, enzyme profile for Laccase, Manganese dependent peroxidase and lignin peroxidase was monitored up to 10 days in presence of 20, 50, 100 and 200 mg/l of all three dyes. Highest laccase specific activity was found 1.58 U/mg in CGY, 1.35 U/mg CNB and 1.43 U/mg in CDR in 20 ppm on 8th day with *P. ostreatus* (Fig. 2). Maximum laccase activity was found in *P. sapidus* was 0.78U/mg in 100 ppm CGY containing flask on 6th day, 0.42U/mg in 20 ppm CNB containing flask on 8th day and 0.5 U/mg in 20 ppm, CDR containing flask on 8th day respectively (Fig. 3). Highest laccase specific activity found in *P. flodida* was 1.68 U/mg in 20 ppm CGY containing flask on 6th day, 1.92 U/mg in 50 ppm CNB containing flask on 10th day and 0.96 U/mg in 20 ppm CDR dye containing flask on 8th day and vey less activity in positive control. There are several reports suggesting role of laccase in dye degradation, various processes has been developed based on laccases due to their potential in degrading dyes of diverse chemical structure (Daâssi et al. 2014; Rodríguez Couto and Toca Herrera 2006). Moreover, the relationship between decolorization efficiency and enzyme activity of white rot fungi was previously reported (Koyani et al. 2013; Niebisch et al. 2014; Ozsoy et al. 2005). Efficient decolorization of dye focused on various factors such as optimization of major medium ingredients, observation of fungal growth, increase in enzyme activity and investigation of decolorization rate (Kaur et al. 2015; Niebisch et al. 2014).

**Fig. 4:**
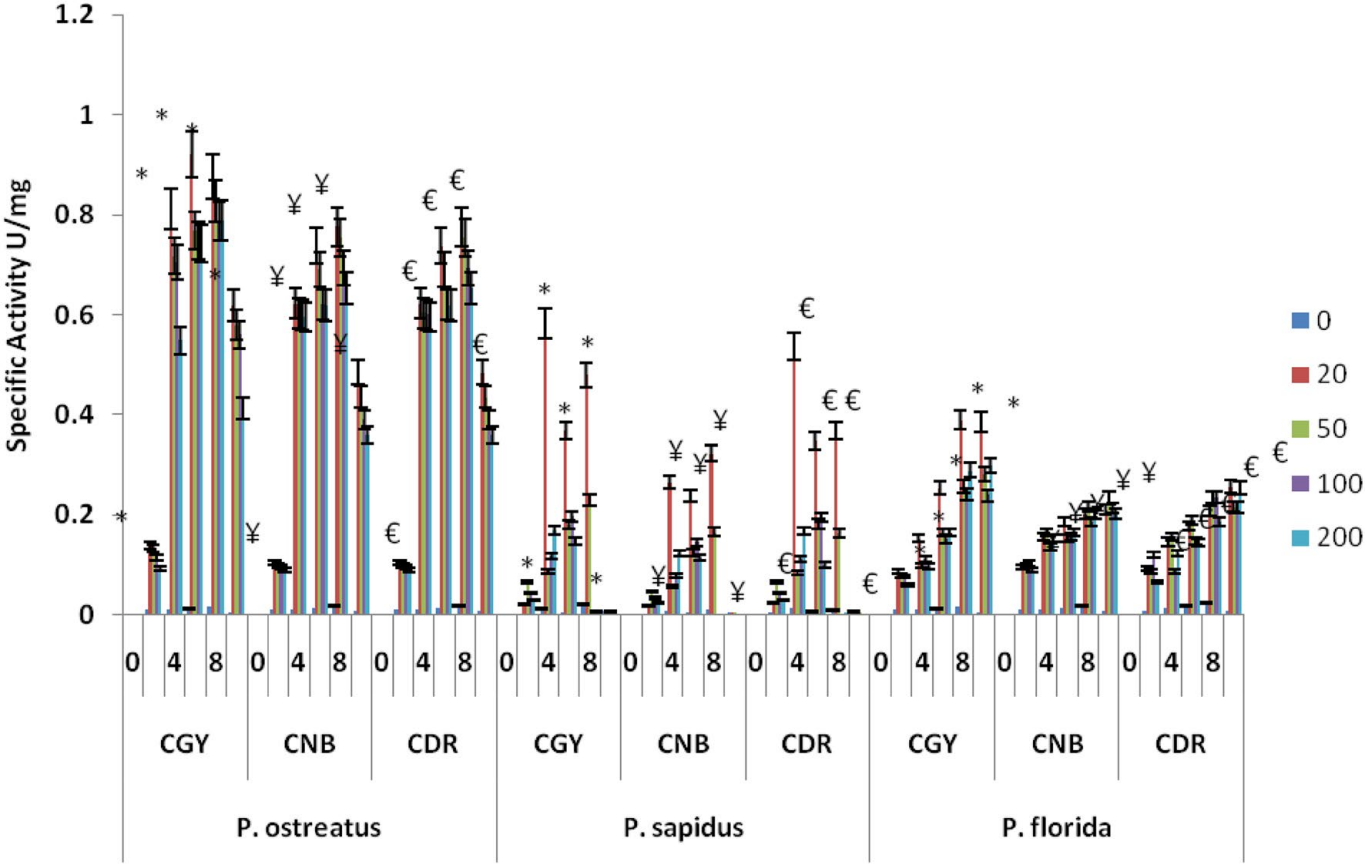
MnP specific activity by *P. ostreatus*, *P. sapidus* and *P. sapidus*: CGY, CNB and CDR. *P < 0.05 compared to control group of CGY, ^¥^ *P* < 0.05 compared to control group of CNB, ^€^ *P* < 0.05 compared to control group of CDR for each respective fungal species

Highest MnP activity found in *P. ostreatus* was 0.88 U/mg in 20 ppm CGY containing flask on 6th day, 0.78 U/mg in 20 ppm, CNB containing flask on 8th day and 0.85 U/mg in 20 ppm, CDR containing flask on 8th day (Fig. 4). In *P. sapidus* maximum MnP was 0.58 U/mg in 20 ppm, CGY containing flask on 4th day, 0.32 U/mg in 20 ppm, CNB containing flask on 4th day, and 0.54 U/mg in 20 ppm CDR containing flask on 4th day and vey less activity in positive control and all other concentrations containing flasks shows maximum MnP enzyme activity in between 0.1 and 0.2 U/mg (Fig. 4). Highest MnP activity found in *P. florida* was 0.389 U/mg in 20 ppm CGY containing flask on 8th day, 0.234 U/mg in 50 ppm CNB containing flask on 10th day and 0.256 U/mg in 20 ppm CDR  containing flask on 10th day and very less activity in positive control (Fig. 4). We have detected MnP and laccase activity in cultures during dye decolorization with significant higher levels of laccase compare to MnP suggesting the important role of Laccase in dye degradation process. However, no LiP activity was found in any of the cultures. Whilst it is clear that enzymes such as MnP, LiP and laccase play a significant role in dye metabolism by white-rot fungi, most interest appears to be the different enzymatic pattern depending on the ligninolytic strains used (Koyani et al. 2013; Pajot et al. 2011; Singh et al. 2013). The no/little LiP activity suggested that the high level of MnP is acting in dye decolorization. MnP enzyme is capable of generating freely diffusible Mn(III) which oxidizes the terminal phenolic substrate, polyphenolics may undergo degradation-dependent binding to the fungal mycelium, and such bound enzymes could be more active than extracellular ones like LiP (Sayadi and Ellouz 1995). Phenolic degradation fragments could serve as substrates of other enzyme systems or be sequestered as osmiophilic granules within the fungal sheath (Sayadi and Ellouz 1995).

### Protein and sugar estimation

Maximum protein concentration was found in all the dyes treated with *P. ostreatus* i.e. 3.5, 3.92, 3.63, respectively in CGY, CNB and CDR after 8 days of inoculation compared to *P. florida* and *P. sapidus,* where very negligible amount of protein was detected i.e. 0.39 mg/ml in case of CNB and 0.35 mg/ml in CGY for later. Results indicated that protein concentration reached maximum at day 8 which supports the maximum enzyme activity for *P. ostreatus*, whereas in case of *P. florida* and *P. sapidus* concentration of protein is fluctuating. Degradation products and/or protein could cause aggregation of dye molecules, preventing the dye uptake to the fabric, which would cause larger color failure (Abadulla et al. 2000).

There are many reports showing the role of sugars especially glucose in dye degradation processes (Radha et al. 2005; Swamy and Ramsay 1999). We found in our study, *P. ostreatus* utilized sugar up to 0.4 and 0.30, 0.35 mg/ml whereas *P. sapidus* and *P. florida* in 20 ppm dye containing flasks. Present results indicate that onset of glucose utilization starts with the fungal growth or initial period of establishment of fungus in dye containing media. As number of day increases, fungi started utilizing dye entities as sole carbon source for its growth and decolorization process, indicated, glucose is not the main active substance in the degradation of azo dyes. Our results supports the earlier findings of Konitou et al. (2002) where increase in concentration of glucose accelerates the process of photocatalyic degradation of dyes. Schiewer and Wong (Schiewer and Wong 2000) reported that color removal from textile effluents increases when glucose is used as co-substrate. It has been proved that removal of 90 and 97 % of Orange G dye using glucose as co-substrate by *P. sordida* and *Tyromyces lauteus*, respectively (Chen et al. 2008).

### Analysis of degradation products

**Fig. 5:**
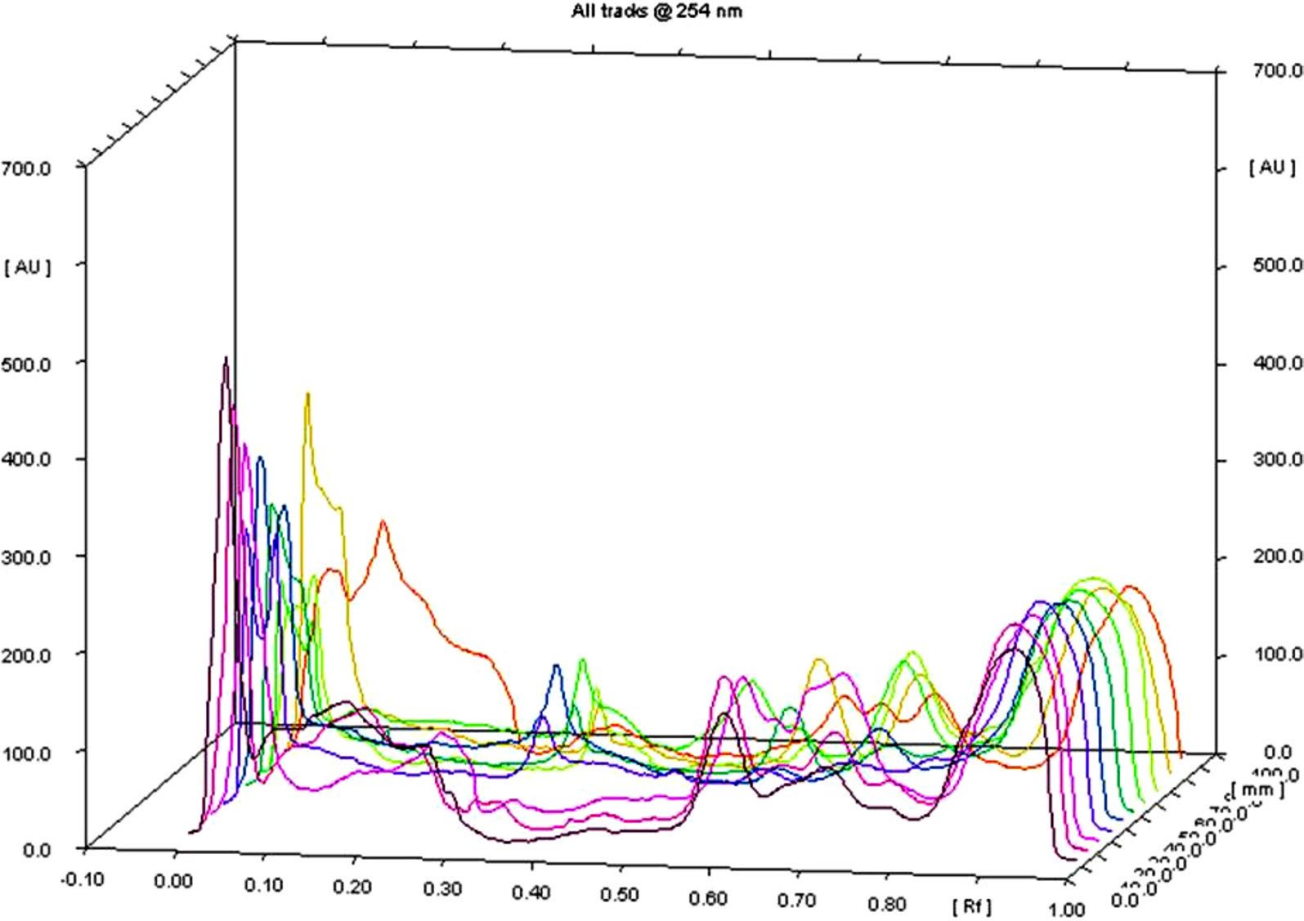
HPTLC analysis at 254 nm

Decolorized dye samples were analyzed from 20 ppm dye containing flask after 10 days, where complete dye decolorization was observed. As shown in Fig. 5 CNB exhibited clear difference in Rf values between decolorized product and control dye in region of 0.07–0.27. *P. sapidus* showed complete elimination of peak from Rf 0.08–0.27 compare to CGY control dye. *P. ostreatus* showed absence of peak in Rf range of 0.70–0.80 for the same dye. The third azo dye CDR, chromatograms of *P. florida* and *P. sapidus* treatment exhibited absence of peaks in Rf region of 0.27 and from 0.07 to 0.27. Detection of new or eliminated peaks as compared to the peaks in control clearly indicated degradation of dye into intermediate products. The analysis of degradation products depends on type of dyes used and its complexity. There are very few findings are available on the biodegradation products or intermediates of different industrial used azo dyes. However, previous studies are performed on reference dyes like degradation of indigo dyes by laccases producing isatin (indole-2,3 dione) which was further degraded to anthranilic acid (2-aminobenzoic acid) detected by HPLC analysis (Balan and Monteiro 2001; Ventura-Camargo and Marin-Morales 2013). In this scenario, futures experiments have been planned to analyze degraded products, which will clear the picture of types of product, evolve after degradation.

## Conclusion

*P. ostreatus* is the best fungal species out of all three studied organism for degradation of azo dyes and linginolytic activity. *P. ostreatus* also exhibits potent medicinal value (Kunjadia et al. 2014), which will help us to design future technologies for production of *P. ostreatus* and ligninolytic enzymes on hazardous dyes for welfare of human kind and biodiversity.

## Abbreviations

ANOVA: Analysis of Variance
CDR: Coralene Dark Red
CGY: Coralene Golden Yellow
CNB: Coralene Navy Blue
HPTLC: High performance thin layer chromatography
LiP: Lignin peroxidase
MnP: Managanese peroxidase
RT: Room temperature
YDA: Yeast dextrose agar
